# Dose Optimization Using a Deep Learning Tool in Various CT Protocols for Urolithiasis: A Physical Human Phantom Study

**DOI:** 10.3390/medicina59091677

**Published:** 2023-09-17

**Authors:** Jae Hun Shim, Se Young Choi, In Ho Chang, Sung Bin Park

**Affiliations:** 1 Department of Urology, Chung-Ang University Hospital, Chung-Ang University College of Medicine, Seoul 06973, Republic of Korea; 2 Department of Radiology, Chung-Ang University Hospital, Chung-Ang University College of Medicine, Seoul 06973, Republic of Korea

**Keywords:** urolithiasis, tomography, X-ray computed, deep learning, radiation dosage, phantoms, imaging

## Abstract

*Background and Objectives:* We attempted to determine the optimal radiation dose to maintain image quality using a deep learning application in a physical human phantom. *Materials and Methods:* Three 5 × 5 × 5 mm^3^ uric acid stones were placed in a physical human phantom in various locations. Three tube voltages (120, 100, and 80 kV) and four current–time products (100, 70, 30, and 15 mAs) were implemented in 12 scans. Each scan was reconstructed with filtered back projection (FBP), statistical iterative reconstruction (IR, iDose), and knowledge-based iterative model reconstruction (IMR). By applying deep learning to each image, we took 12 more scans. Objective image assessments were calculated using the standard deviation of the Hounsfield unit (HU). Subjective image assessments were performed by one radiologist and one urologist. Two radiologists assessed the subjective assessment and found the stone under the absence of information. We used this data to calculate the diagnostic accuracy. *Results:* Objective image noise was decreased after applying a deep learning tool in all images of FBP, iDose, and IMR. There was no statistical difference between iDose and deep learning-applied FBP images (10.1 ± 11.9, 9.5 ± 18.5 HU, *p* = 0.583, respectively). At a 100 kV–30 mAs setting, deep learning-applied FBP obtained a similar objective noise in approximately one third of the radiation doses compared to FBP. In radiation doses with settings lower than 100 kV–30 mAs, the subject image assessment (image quality, confidence level, and noise) showed deteriorated scores. Diagnostic accuracy was increased when the deep learning setting was lower than 100 kV–30 mAs, except for at 80 kV–15 mAs. *Conclusions:* At the setting of 100 kV–30 mAs or higher, deep learning-applied FBP did not differ in image quality compared to IR. At the setting of 100 kV–30 mAs, the radiation dose can decrease by about one third while maintaining objective noise.

## 1. Introduction

With a 10.6% male prevalence rate and a 7.1% female prevalence rate, urolithiasis is a widespread disease [1]. Although the prevalence differs according to country, the incidence and prevalence are rising due to increasing obesity or systemic disease rates and social situations [2]. Around 10% of patients experience numerous recurrences, while approximately 50% of recurrent cases experience just one [3,4]. For these patients, thin section non-enhanced computed tomography (CT) has been used frequently because it offers a quick, precise evaluation [5]. In this case, additional evaluation is performed to confirm the presence or absence of stones. CT, which can identify stones by checking the surrounding anatomical structures, does not have a delay time and is increasingly being used [3,4,5].

The typical radiation dose of standard or regular dose CT for stone disease is 8–10 mSv and the exposed amount of the dose could be increased according to the organ or field [3,4]. The International Commission on Radiological Protection recommended a radiation dose limit of up to a mean of 20 mSv per year [6]. The potential carcinogenic effect of CT scans is concerning [7,8]. Urinary tract stones are prone to recurrence and patients are likely to be exposed to radiation with repeated CT scanning [3,4,9]. Low-dose CTs may reduce the risk of radiation exposure. Recently, the advent of ultra-low dose CT (ULDCT) has led to an extremely low level of radiation exposure (<1.9 mSv), compared to both the low-dose CT (LDCT) at 3 mSv and the standard X-ray at 2.15 mSv [10]. A similar result was demonstrated in another study, where radiation exposure was 1.28 ± 0.34 mSv for ULDCT vs. 5.49 ± 1.00 mSv for LDCT, without altering the examination’s detection rate [11]. However, trying to minimize the radiation exposure, low-dose CT results in low image quality due to increased noise [12]. For patients with urinary stones, image noise reduction is crucial to enabling scanning at a reduced radiation dosage.

With the recent advancement of artificial intelligence, radiology integration is progressing quickly. Deep learning of a convolutional neural network (CNN) has had a significant impact on diagnostic image analysis in urology [13]. We aimed to determine if deep learning could help reduce the radiation risk in urinary tract stones.

## 2. Materials and Methods

### 2.1. Phantom Model

**Figure 1:**
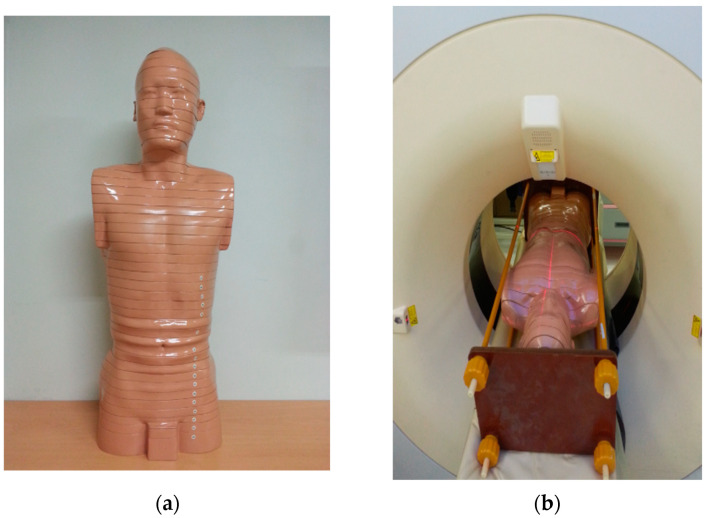
Model of the Korean physical phantom. (**a**) The phantom has 43 slices, each with a thickness of 2 cm. (**b**) The phantom was fixed in a supine position in a frame to prevent shaking during CT scanning.

For this investigation, the Radiation Health Research Institute of Korea Hydro and Nuclear Power (Seoul, Republic of Korea), provided the standard Korean male physical phantom (Figure 1a). The phantom size weighs 68 kg, stands 172 cm tall, and has a body mass index of 22.99 kg m^2^. The model simulates the average height and weight of a Korean male. Epoxy resin, urethane foam, and polyurethane were used to create the phantom’s bone, lungs, and soft tissues, and they had densities that were comparable to that of human tissue. The phantom was cut into 2 cm thick slices. It had grids with holes that were 7 mm in diameter and spaced 2 cm apart. We placed an approximately 5 × 5 × 5 mm^3^ uric acid stone in a hole. In the phantom model, we determined that a low CDIvol value could affect the subjective evaluation of stones near the epoxy resin. To improve the accuracy of the subjective evaluation, we placed the three stones in various locations (proximal, mid, and distal based on the locations of frequent ureteral stones symptoms). To avoid movement during scanning, the phantom was locked in a supine posture in a frame (Figure 1b).

### 2.2. CT Protocol and Image Reconstruction

A 256-multidetector CT scanner (Brilliance iCT; Philips Healthcare, Cleveland, OH, USA) was used to scan the phantom. Standard non-enhanced CT for urolithiasis was performed with a scan range between the proximal aspect of the 12^th^ thoracic vertebra and the distal aspect of the symphysis pubis of the phantom in the supine position. For all scans, an automated z-axis dose modulation (DoseRight; Philips Healthcare) derived from the survey image was used. The following were the scanning parameters: kernel, B(standard) filer; section thickness, 3 mm; increment, 2.7 mm; detector configuration, 128 × 0.625; pitch, 0.915; beam collimation, 80 mm; rotation time, 0.4 s; and helical acquisition. Four current–time products (100, 70, 30, and 15 mAs) and three tube voltages (120, 100, and 80 kV) were employed. We had 84 reconstructed imaging data from a previous study (FBP, iDose 4 (Levels 5–7) and IMR (soft tissue Levels 1–3)) [14]. For this study, ClariCT was applied to each CT image. ClariCT (ClariPi, Seoul, Republic of Korea) is based on the CNN algorithm noise reduction approach and features digital imaging and communications in medicine (DICOM)-based sinogram blend and statistical iterative reconstruction (IR). It has benefits in terms of denoising from both projection and image space. A total of 168 reconstructed imaging data sets (84 pre-processed through the various reconstruction and 84 post-processed using ClariCT) were obtained.

### 2.3. Radiation Dose

The manufacturer’s CT scanner software recorded the volume CT dose index (CTDIvol) and dose–length product (DLP). The ED was calculated from the DLP using a constant region-specific normalized effective dose (ED) value of 0.015 mSv^−1^ mGy^−1^ cm. Size-specific dose estimate (SSDE) values were used to consider the patient size.

### 2.4. Objective Image Quality Assessment

**Figure 2:**
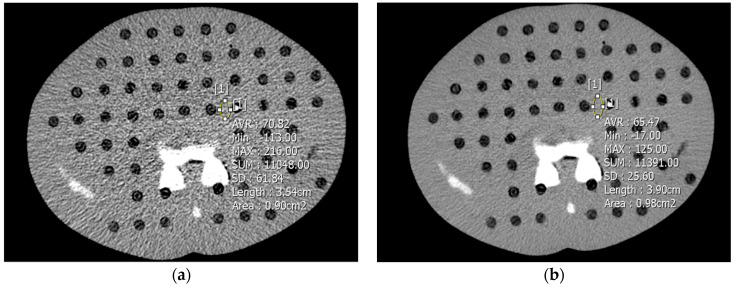
The representative CT image of the phantom at 100 kV and 30 mAs. The noise in the images was produced by filtered back projection (FBP) (a) and FBP applied ClariCT (b). The objective image noise was evaluated as the standard deviation of the Hounsfield unit in round legions (each with an area of 1 cm^2^).

An independent urologist who was not involved in the subjective image evaluation reviewed the objective image noise. The Hounsfield unit (HU) standard deviation in circular region of interest, each with a 1 cm^2^ area, were put in the homogenous soft tissue of the phantom around the distal ureter stone allocated to them (Figure 2). The CT number (HU) divided by the standard deviation was used to define the signal-to-noise ratio (SNR).

### 2.5. Subjective Image Assessment

One radiologist and one urologist, both with at least ten years of expertise, independently and subjectively reviewed the 168-image data set while being unaware of the scan settings or reconstruction techniques. We evaluated the average of the readers’ results. All images were transmitted to picture archiving and communication systems and displayed randomly on a Barco monitor that was calibrated every three months with s resolution of 1200 pixels wide and 1600 pixels high. The default window/level was 400/40, however this may be altered as needed. On a five-point scale, the image quality surrounding the target stone that was inserted into the hole was rated (1 = poor image quality, not diagnostically acceptable for interpretation; 2 = suboptimal image quality, worse than acceptable quality; 3 = acceptable image quality, diagnostic interpretation possible; 4 = good image quality; and 5 = excellent image quality). On a three-point scale, the level of confidence in the urolithiasis diagnosis was graded (1 = no confidence; 2 = confidence with reservations; and 3 = highly confident). Using the image’s graininess or pixel-to-pixel variance, a three-point scale was used to subjectively rate the noise in the entire image (1 = minimal; 2 = acceptable; and 3 = excessive, rendering diagnostic interpretation impossible).

### 2.6. Subjective Stone Diagnosis Assessment

The stone was placed somewhere in the phantom. The stone detectability was evaluated by 2 staff radiologists blinded to the presence of the stones. They did not perform the subjective image assessment and evaluated by consensus. The overall diagnostic acceptability for each stone was graded on a three-point scale (1 = poor image quality, not diagnostically acceptable for interpretation, 2 = suboptimal image quality, worse than acceptable quality, and 3 = diagnostic interpretation possible). The radiologists found the stone (1 = found and 0 = not found or wrong).

### 2.7. Statistical Analyses

The paired *t*-test and Wilcoxon signed-rank test were used to compare the objective image noise, SNR, subjective image quality, confidence level, and image noise from filtered back projection (FBP), statistical IR (iDose), and knowledge-based iterative model reconstruction (IMR) data, with ClariCT applied to each. SPSS^®^ v. 21.0 (IBM Corp., New York, NY, USA; formerly SPSS Inc., Chicago, IL, USA) was used for all statistical analyses. Significance was set at *p*-value < 0.05.

To subjectively assess the stone diagnosis, we calculated the stone cut-off value using the receiver operating characteristic curve according to the overall diagnostic acceptability grade. Based on the cut-off value, we divided the images into two groups and matched the group that answered correctly and the group that answered incorrectly or did not answer. Diagnostic accuracy was calculated as TP/(TP + FP + TN + FP) × 100 [true positive (TP), true negative (TN), false positive (FP), and false negative (FN)].

## 3. Results

### 3.1. Radiation Dose

Table 1 provides an overview of CT scan settings and dosage data. Lower voltages and current–time products tended to display lower CTDI_vol_, SSDE, DLP, and ED. The EDs were between 0.095–2.621 mSv.

### 3.2. Quantitative Analysis of the Image Quality

**Figure 3:**
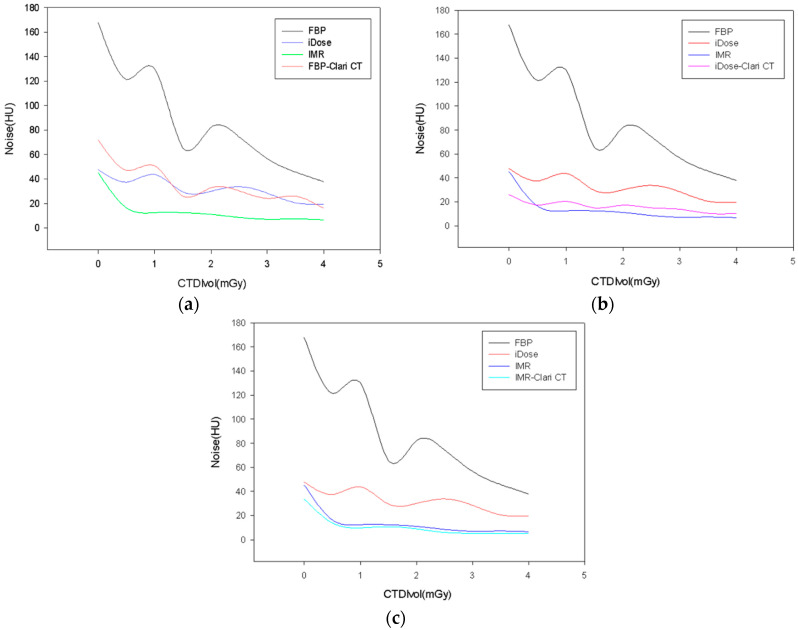
Relationship between objective image noise and CTDIvol. (**a**) Objective image noise of FBP, iDose, IMR, and FBP applied ClariCT. (**b**) Objective image noise of FBP, iDose, IMR, and iDose applied ClariCT. (**c**) Objective image noise of FBP, iDose, IMR, and IMR applied ClariCT.

**Figure 4:**
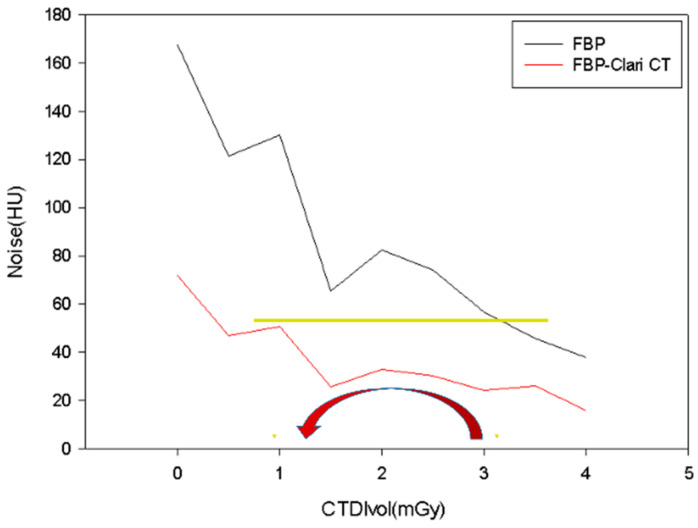
Relationship of objective image noise between FBP and FBP applied ClariCT. When the amount of noise is almost the same, the FBP applied ClariCT decreases the radiation by 70% compared to FBP.

As seen in Figure 3, the connection between CTDI_vol_ and the objective image noise was inverse. FBP and ClariCT-applied FBP had significantly different objective noise levels (18.1 ± 46.8 and 9.5 ± 18.5 HU, respectively, *p* = 0.002) (Figure 3a), as did iDose and ClariCT applied iDose (10.1 ± 11.9 and 6.6 ± 5.7 HU, respectively, *p*= 0.002) (Figure 3b). IMR and ClariCT applied IMR also had significantly different objective noise levels (4.4 ± 11.1 and 3.5 ± 8.3 HU, respectively, *p* = 0.002) (Figure 3c). Interestingly, there was no statistically significant difference in the levels of iDose and ClariCT-applied FBP (10.1 ± 11.9 and 9.5 ± 18.5 HU, respectively, *p* = 0.583). On the other hand, when the noise value was the same, the amount of radiation could be reduced by approximately one-third, in the 120 kV–15 mAs setting (Figure 4). However, there was a slightly statistically significant difference in the levels of IMR and ClariCT applied iDose (4.4 ± 11.1 and 6.6 ± 5.7 HU, respectively, *p* = 0.034, respectively).

### 3.3. Qualitative Analysis

**Figure 5:**
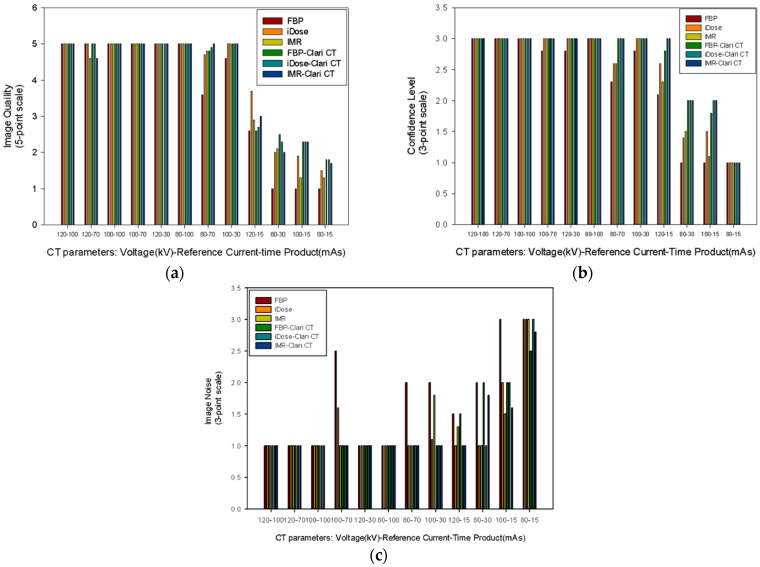
Subjective image assessment by each CT parameter. (**a**) Image quality on a five-point scale. (**b**) Confidence level on a three-point scale. (**c**) Subjective image noise on a three-point scale.

In the case of an ED greater than 0.431 mSv (100 kV–30 mAs setting), there was no difference in the subjective evaluation of all three graphs (Figure 5). There was a statistical difference in the subjective score at settings between ED 0.372 mSv (120 kV–15 mAs) and ED 0.431 mSv (100 kV–30 mAs), including the image quality, confidence level, and subjective image noise. The FBP image quality score was 2.6 in the 120 kV–15 mAs setting and 4.6 in the 100 kV–30 mAs setting (*p* = 0.083). The ClariCT applied FBP image quality score was 2.6 in the 120 kV–15 mAs setting and 5.0 in the 100 kV–30 mAs setting (*p* = 0.102) (Figure 5a). The FBP confidence level was 2.1 in the 120 kV–15 mAs setting and 2.8 in the 100 kV–30 mAs setting (*p* = 0.102). In the 120 kV–15 mAs setting, the ClariCT applied FBP confidence level was 2.8, while in the 100 kV–30 mAs setting, it was 3.0 (*p* = 0.317) (Figure 5b). The FBP subjective image noise score was 1.5 in the 120 kV–15 mAs setting and 2.0 in the 100 kV–30 mAs setting (*p* = 0.317). In the 120 kV–15 mAs setting, the ClariCT applied FBP subjective image noise score was 1.5, while in the 100 kV–30 mAs setting, it was 1.0 (*p* = 0.317) (Figure 5c). There were no differences between iDose and ClariCT applied iDose in the image quality (*p* = 0.498), confidence level (*p* = 0.066), and subjective image noise (*p* = 0.180). Likewise, there were no differences between IMR and ClariCT applied IMR in the image quality (*p* = 0.104), confidence level (*p* = 0.068), and subjective image noise (*p* = 0.588).

The break point of the subjective image quality, confidence level, and image noise is 0.63 mGy in CTDIvol.

When the CTDIvol was >0.54 (120 kV–15 mAs setting), there was little difference in subjective evaluation and ability to diagnose stones. However, when the CTDIvol was <0.54, there was a difference depending on the image reconstruction method and whether ClariCT was applied.

### 3.4. Diagnostic Accuracy for Stone Detection

The cut-off value of the overall diagnostic acceptability grade was 1.5. The diagnostic accuracy is shown in Table 2. In the 120 kV–15 mAs setting, the CTDIvol value was 0.54 with FBP reconstruction, and the accuracy increased from 65 to 75 when ClariCT was applied. This approximates the accuracy of iDose (*p* = 0.083). Overall, the accuracy increased when ClariCT was applied at a low CTDIvol, except for at the 80 kV–15 mAs setting (Table 2).

## 4. Discussion

This study compared a deep learning image reconstruction (DLIR) algorithm with FBP, iDose, and IMR algorithms for the reconstruction of urolithiasis CT. We evaluated the image quality (objective and subjective) of different CT protocols and reconstructions and urinary stone diagnostic accuracy. DLIR improved noise characteristics and image quality. ClariCT results in better-quality images with less radiation. Applying ClariCT lowers radiation dose during CT scans, which can lower radiation exposure. As the subjective assessment score shows a significant decrease when it is less than 100 kV–30 mAs setting, image quality improves if ClariCT is applied. FBP is the most widely used method, and the amount of radiation exposure may be high to obtain a quality image [15]. In this study, applying DLIR to FBP showed objective noise similar to iDose with a one-third reduction in radiation. As there was no difference in subjective assessment, applying ClariCT to iDose or IMR has insignificant effect. Therefore, applying ClariCT to CT images using FBP will have the greatest noise reduction. Also, deep learning denoising techniques are vendor agnostic; that is, they can be applied regardless of vendor and regardless of reconstruction technique. In other words, their great advantage is that they can reduce the amount of radiation to which patients are exposed and improve the quality of images without adding new vendor.

This study was conducted under a 2.621 mSv (120 kV–100 mAs) setting or less, which is lower than the radiant dose of conventional CT conditions. This confirms it is possible to check for stones at a low radiation dose using ClariCT.

Low-dose radiation is defined as less than 3.5 mSv and ultra-low-dose as less than 1.9 mSv [16]. In our study, the ED for 100 kV–30 mAs setting was 0.431 mSv, which is equivalent to an ultra-low dose. The ED of CT, which is mainly taken with urinary stones, exceeds about 3 mSv [17]. This deep learning program can maintain the image quality of stone reading with radiation values corresponding to an ultra-low dose. FBP is a widely used reconstruction method in many hospitals [18] and requires no special additional hardware. Statistical IR (iDose) and model-based IR (IMR) require hardware from the manufacturers, making them less versatile and more expensive [19,20].

In this study, the optimal setting for urolithiasis is 100kV–30 mAs more specifically, in terms of objective image noise, subjective image quality, diagnostic accuracy, and use of deep learning reconstruction technique.

Attempts to apply deep learning are also being actively made in the field of urinary tract stones [13]. Deep learning can be used to analyze the components of the stone using only endoscopic images of urolithiasis [21,22]. Kidney stones can also be found using deep learning in the CT images [23]. This model was 96% accurate when compared with two experts [23]. In addition, deep learning has been applied to low-dose or ultra-low-dose CT denoising. Despite the significant dose reduction achieved with ultra-low dose CT, diagnostic performance for urolithiasis remained excellent using two vendor-specific deep learning imaging reconstruction algorithms (TrueFidelity in GE Healthcare and Advanced intelligent Clear-IQ Engine in Cannon Medical System) [24]. It was consistent with this study in terms of deep learning imaging reconstruction. However, this is the first study to use vendor neutral deep learning image reconstruction algorithm.

There is no evidence to suggest that values below 1 mSv can prevent radiation-induced cancer transformations. But a recent meta-analysis revealed a significant correlation between CT scan and cancer risk and said that cancer is more likely to occur in the area where CT is taken [25]. The less exposure to radiation, the lower the probability of cancer occurrence, so we should make efforts to ensure that there is less radiation continuously exposed. Furthermore, C-arm X-rays are often used for endourological procedures, and endourological procedures can frequently expose operators and patients to radiation. So, we should effort to reduce the amount of radiation that directly reaches the human body by wearing protective gear while taking minimal C-arm X-ray images [26].

Patients with frequent recurrence of urinary tract stone or high risk for urinary tract stone formation often need to undergo non-enhanced CT multiple times. Urologists gathered to publish their papers in FAQ format, and it is recommended that patients with recurrent stones or high-risk groups for the Asian population take images every three to six months [27]. Applying deep learning techniques to real clinical setting would be good for both doctor and patient. On the patient side, the more radiation exposure is minimized, the more helpful it will be for the patient. On the doctor side, this is because it helps read by reducing image noise.

Our study has several limitations. First, in clinical practice, patients of various sizes may have stones of different positions, compositions, and sizes in their bodies. The optimal radiation dose should be set to ensure the detection of stones with all common components, sizes, and positions in most people of body types, instead of detecting stones with a single composition and single size in patients of standard size. Further research needs to at least count the characteristics of stone patients in a single center, including the thickness of the abdominal fat layer and the data of stone location, composition, and size, and select a reasonable test range. Also, BMI is an important factor affecting CT reading and should be considered when determining optimal radiation dose of low dose CT. In the future, it is thought that follow-up studies will be possible if Korean female phantom models are developed or BMI is applied to create phantom of various body types. Second, this study was not modeled on humans, but on a phantom copy of the human body without the organs. This study required the use of a phantom to determine the radiation dose as it is unethical to repeatedly scan humans by applying various radiation settings. However, no matter how similar a phantom is to the human body, it will not be identical. It is inevitable, inherent, and hard to correct. Third, the experiment was conducted with knowledge regarding stone location. Since the results were predicted and evaluated, the subjective score could be quite high. Other test results, such as clinical symptoms and laboratory data, may differ from experimental phantom studies because they aid in making a diagnosis. Future research involving actual patient data is warranted. However, we have confirmed that our research can reduce objective noise indicators. Fourth, only uric acid stones were considered in this study. We will proceed with the follow-up study to see if there is any change in the optimal radiation dose to maintain image quality using a deep learning application in a physical human phantom when we conduct research on stones of other components including calcium phosphate. Furthermore, in the real clinical practice, the size, shape, and location of the urolithiasis has large spectrum. Finally, we did analyze other objective methods, such as the structural similarity index and edge rise distance [28,29].

## 5. Conclusions

In conclusion, we suggest an optimal imaging setting at 100 kV and 30 mAs for urolithiasis in a Korean phantom model. When a deep learning is FBP, the noise was similar to IR. Deep learning could reduce the radiation dose to approximately 70% compared to the same amount of FBP noise. It is said that an accurate diagnosis can be made by reducing the amount of radiation to which patients are exposed when diagnosing stones. In the future, there will be a strong tendency to use artificial intelligence as diagnostic assistance, reduce noise, and further predict the success rate of treatment through individual customized treatment.

## Figures and Tables

**Table 1:** Scan parameters and dose reports.

Voltage (kV)	Reference Current–Time Product (mAs)	Effective Current–Time Product (mAs)	CTDI_vol_ (mGy)	SSDE (mGy)	DLP (mGy)	ED (mSv)
120	100	84	3.82	5.73	174.7	2.621
120	70	44	2.7	4.05	123.4	1.851
120	30	19	1.11	1.665	50.9	0.764
120	15	10	0.54	0.81	24.8	0.372
100	100	61	2.25	3.375	102.9	1.544
100	70	43	1.56	2.34	71.3	1.07
100	30	18	0.63	0.945	28.7	0.431
100	15	10	0.28	0.42	13	0.195
80	100	58	1.03	1.545	46.9	0.704
80	70	41	0.72	1.08	33	0.495
80	30	18	0.3	0.45	13.8	0.207
80	15	10	0.14	0.21	6.3	0.095

CTDI_vol_, volume computed tomography (CT) dose index; SSDE, size-specific dose estimate; DLP, dose length product; and ED, effective dose.

**Table 2:** Diagnostic accuracy for stone detection.

CTDI_vol_ (mGy)	Voltage (kV)	Reference Current–Time Product (mAs)	FBP	iDose	IMR	FBP-ClariCT	iDose-ClariCT	IMR-ClariCT
3.82	120	100	100	100	100	100	100	100
2.7	120	70	100	100	100	100	100	100
2.25	100	100	100	100	100	100	100	100
1.56	100	70	100	100	100	100	100	100
1.11	120	30	100	100	100	100	100	100
1.03	80	100	100	100	100	100	100	100
0.72	80	70	100	100	100	100	100	100
0.63	100	30	100	100	100	100	100	100
0.54	120	15	65	80	75	75	85	85
0.3	80	30	5	35	50	65	65	65
0.28	100	15	10	35	55	55	55	60
0.14	80	15	0	0	0	0	0	0

Data presented as percentage (%). CTDI_vol_, volume computed tomography (CT) dose index.

## Data Availability

Not applicable.

## Abbreviations

CT	computed tomography
FBP	filtered back projection
IR	iterative reconstruction
IMR	iterative model reconstruction
HU	Hounsfield unit
IVP	intravenous pyelography
CNN	convolutional neural network
DICOM	digital imaging and communications in medicine
DLP	dose–length product
CTDIvol	volume CT dose index
ED	effective dose
SSDE	size-specific dose estimate
SNR	signal-to-noise ratio
